# Incidence of epicardial connections between the right pulmonary vein carina and right atrium during catheter ablation of atrial fibrillation: A comparison between the conventional method and unipolar signal modification

**DOI:** 10.1002/joa3.12672

**Published:** 2021-12-27

**Authors:** Hiroki Yano, Taku Nishida, Junichi Sugiura, Ayaka Keshi, Koshiro Kanaoka, Satoshi Terasaki, Yukihiro Hashimoto, Yasuki Nakada, Hitoshi Nakagawa, Tomoya Ueda, Ayako Seno, Kenji Onoue, Makoto Watanabe, Yoshihiko Saito

**Affiliations:** ^1^ Department of Cardiovascular Medicine Nara Medical University Kashihara Japan

**Keywords:** atrial fibrillation, carina, epicardial connection, high‐power, unipolar signal modification

## Abstract

**Background:**

When performing an electrical isolation of ipsilateral pulmonary veins (PVs) for atrial fibrillation, physicians often need additional radiofrequency (RF) ablation in the carina region between the superior and inferior PVs to achieve a right PV isolation because of intercaval bundles between the right PVs and right atrium (RA). We compared the efficacy of a high‐power and short‐duration ablation guided by unipolar signal modification (UM) with the conventional method (CM) for ablating epicardial connections between the right PV carina and RA.

**Methods:**

The study subjects consisted of patients who underwent an initial box isolation of atrial fibrillation from January 2015 to December 2019 at Nara Medical University Hospital. Among these patients, 94 and 65 patients who met the criteria were assigned to the CM and UM groups, respectively. We retrospectively analyzed the anterior ablation line of the right PV using an electroanatomical mapping system. Patients whose initial ablation line included the right PV carina were excluded.

**Results:**

Six and seven patients were, respectively, excluded from the CM and UM groups. Among 88 CM group patients, 21 needed additional right PV carina ablation, while among 58 UM group patients, 30 needed additional right PV carina ablation (*p* = .001). No anatomical factors were associated with the additional right PV carina ablation.

**Conclusions:**

Compared to the CM group, a box isolation was less achievable without RF ablation at the right PV carina in the UM group. We should consider a long‐duration ablation for epicardial connections between the right PV carina and RA.

## Graphical abstract

The safety and efficacy of a high‐power (40–50 W) ablation using unipolar signal modification as a local endpoint has been reported. There have been some reports about epicardial connections between the pulmonary veins and right atrium (RA). A box isolation was less achievable without additional radiofrequency ablation on the right PV carina in a high‐power and short‐duration ablation guided by unipolar signal modification group than in the conventional long‐duration ablation group.

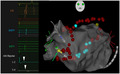

## 1 INTRODUCTION

Electrical isolation of the pulmonary veins (PVs) is recommended during atrial fibrillation (AF) ablation procedures.^1^ A transmural lesion formation is essential to achieve a PV isolation. However, epicardial connections are one of the anatomical factors that sometimes preclude a PV isolation. There have been some reports about epicardial connections between the PVs and right atrium (RA),^2^ ipsilateral PVs,^3^ or PVs and the left atrium (LA).^1^

The abolition of the negative component of the unipolar signal, which reflects the lesion transmurality, has been reported in both experimental and clinical studies,^4^, ^5^ where radiofrequency (RF) energy is applied with a conventional low‐power (25–30 W) and long‐duration setting. Recently, the safety and efficacy of a high‐power (40–50 W) ablation using unipolar signal modification as a local endpoint has also been reported.^6^ In swine models, collateral damage to neighboring structures that often forms because of conductive heating is reduced by the high‐power short‐duration ablation.^7^, ^8^, ^9^ Less collateral damage leads to fewer complications. In contrast, less collateral damage might lead to insufficient epicardial lesions. The presence of epicardial connections between the PVs and other anatomical structures might make the PV isolation more difficult.^10^ In patients with epicardial connections between the right PVs and RA, physicians often need to apply RF ablation in the carina region between the superior and inferior PVs to achieve a right PV isolation. In addition, we previously reported a case who required an RF ablation on the RA posterior wall adjacent to the septum to achieve a box isolation because of an epicardial connection between the right PV carina and RA.^11^

We hypothesized that a high‐power and short‐duration ablation using unipolar signal modification might lead to failure of achieving a complete right PV isolation because of epicardial connections between the right PVs and RA. In this study, we compared the conventional ablation with the high‐power and short‐duration ablation using unipolar signal modification during the right PV isolation.

## 2 METHODS

### 2.1 Study subjects

Ninety‐four and sixty‐five patients who underwent a box isolation as an initial catheter ablation of AF and met the criteria from January 2015 to December 2019 at Nara Medical University Hospital were retrospectively included in this study. Patients without three‐dimensional (3D) mapping data or whose initial ablation line was applied to the carina region were excluded. All antiarrhythmic drug therapies were discontinued five half‐lives before the procedure except for amiodarone. All patients received oral anticoagulants for at least 1 month before the procedure, and transesophageal echocardiography or contrast‐enhanced cardiac computed tomography was performed to exclude any LA thrombi before the procedure. The clinical, anthropometric, and anatomical variables were collected from all patients. The AF‐related variables included the type of AF, that is, paroxysmal, persistent, or long‐standing AF. Patients with structural heart disease were divided into old myocardial infarction, coronary artery disease, nonischemic cardiomyopathy, valvular heart disease, and tachycardia‐induced cardiomyopathy groups. When the ventricular dysfunction was attributable to AF, it was considered a tachycardia‐induced cardiomyopathy. Echocardiography was performed in all patients to obtain information on the left ventricular ejection fraction, LA size, and valvular disease. Cardiac computed tomography was also performed in all patients except for those with severe renal dysfunction or allergies to contrast medium. The LA volume and right PV configuration were analyzed. The right PV configuration was divided into two types according to the number of right PVs: two versus three or more.

The study protocol was approved by the hospital's institutional review board and complied with the Declaration of Helsinki.

### 2.2 Electrophysiological study and catheter ablation

An electrophysiological study and catheter ablation were performed under general anesthesia with propofol and fentanyl. Before sedation, the patients were asked to swallow 5 ml of contrast, and the location of the esophagus was noted on fluoroscopy. The surface electrocardiograms and intracardiac electrograms were continuously monitored and stored on an EP‐WorkMate (Abbott) or RMC‐5000 (Nihon Kohden). Three‐dimensional mapping systems (CARTO; Biosense Webster or EnSite; Abbott) were used in all patients. The intracardiac electrograms were filtered from 30 to 300 Hz and measured at a sweep speed of 100 or 150 mm/s. Atrial or ventricular pacing was performed using a programmed stimulator (SEC‐5104; Nihon Kohden or BC‐1100; Fukuda Denshi). After vascular access, 60 IU heparin/kg was given intravenously, and heparinization was continued during the procedure to maintain an activated clotting time higher than 300 s. A 6F 20‐pole mapping catheter (BeeAT; Japan Lifeline) was positioned in the coronary sinus through the internal jugular vein. An intracardiac echocardiography catheter (AcuNav; Biosense Webster or Ultra ICE IntraCardiac Echo Catheters; Boston Scientific) was advanced into the RA via the femoral approach to guide the transseptal puncture. Three long sheaths (Agilis Nxt Steerable Introducer, SL0 8Fr, and SL0 8.5Fr Swartz Braided Sheath; Abbott) were then advanced into the LA. We preferentially performed a box isolation as an initial RF ablation strategy of AF. However, if both boarders of the esophagus were between the left and right inferior PVs, we performed a PV isolation to avoid direct RF applications adjacent to the esophagus. We performed a complete isolation of the posterior LA including the four PVs with an irrigated‐tip catheter (Biosense Webster ThermoCool SmartTouch or ThermoCool STSF; Abbott Therapy Cool Flex or FlexAbility). In the conventional method (CM) group, RF energy was delivered at 30 W for 30 s except for at sites adjacent to the esophagus where the RF energy was reduced to 25 W. The RF application was prolonged over the 30 s at sites with catheter instability at the discretion of the operator. The RF applications were terminated if the temperature recorded by the esophageal probe rose to 39.0°C. In the high‐power and short‐duration ablation guided by unipolar signal modification (UM) group, RF energy was delivered generally with 40 and 30 W at sites near the esophagus. The local endpoint of each RF application was the abolition of the negative component of the local unipolar signal. Each RF application lasted for 5 to 10 s after the change in the unipolar signal. After each RF application, the ablation catheter was moved until a new positive‐negative unipolar signal was recorded, constituting the next ablation site. The applications near the esophagus were stopped if the temperature of the esophageal probe rose to 39.0°C. The procedural endpoint was bidirectional block of the box lesion. If the box isolation was not achieved by the circumferential ablation, careful mapping to identify any gaps with a circular mapping catheter or the ablation catheter was performed, and additional RF applications were repeated to eliminate the residual potentials. If the right PV carina ablation resulted in a successful box isolation, this application at the carina was considered indispensable. We monitored the PV potentials for 60 min from the initial documentation of the box isolation. If the PVs remained isolated, we administered adenosine triphosphate to detect any dormant conduction, except if asthma or severe coronary heart disease was suspected. If dormant PV conduction was identified, additional RF energy was applied to establish a complete box isolation. In the present study, when we had to apply RF energy at either the carina region, apart from the initial ablation line or posteroseptal wall of the RA, we considered there were epicardial connections between the right PV carina and RA. Additionally, we also analyzed other epicardial connections: the septopulmonary bundle running vertically along the posterior wall of LA, or connections between the left PVs and LA through the ligament of Marshall. When additional RF applications were needed at the posterior side of the roof line or inside the initial anterior line of the left PVs, we considered epicardial connections, respectively.

The data from the EP‐WorkMate (Abbott) or RMC‐5000 (Nihon Kohden) were analyzed to calculate the number of RF applications and total and average RF application time. These RF parameters along the anterior aspect of the right PVs between the top and bottom of the right PVs were also specifically analyzed. In cases using a CARTO system (Biosense Webster), the VisiTag data were used to analyze the duration of the RF applications on a point‐by‐point basis.

### 2.3 Follow‐up

After discharge, all patients underwent a 24‐h ambulatory monitoring every 3 months for 1 year. In case of symptoms suggesting an arrhythmia recurrence, patients were advised to have an additional 12‐lead electrocardiogram recorded or undergo additional 24‐h ambulatory monitoring or patient‐driven event monitoring (HCG‐801, Omron Healthcare Co.). Atrial arrhythmia recurrence was defined by any documented episode of AF or atrial tachycardia lasting more than 30 s after a 3‐month blanking period from the ablation procedure.

### 2.4 Statistical analysis

Continuous variables are expressed as the mean ± standard deviation (SD) or median (25th–75th percentile) and categorical variables as the number and percentage. Differences between groups were tested using the unpaired student *t*‐test, Mann–Whitney *U*‐test, Pearson's chi‐square analysis, Fisher's test, or adjusted standardized residuals as appropriate. A two‐tailed *p* value lower than .05 indicated statistical significance. We used multiple imputation to handle any missing data. All statistical analyses were carried out using R software (the University of Auckland).

## 3 RESULTS

### 3.1 Patient characteristics

In the CM group, six patients were excluded because there were no 3D mapping data in two, and the initial ablation lines were applied on the right PV carina in four. In the UM group, seven patients were excluded because no 3D mapping data was available in one, and the initial ablation lines were applied on the right PV carina in six. Finally, 88 patients were included in the CM group and 58 in the UM group. There were no significant differences in the age, body mass index, hypertension, diabetes mellitus, and chronic kidney disease between the CM and UM group patients. The CHA2DS2‐VASc score, brain natriuretic peptide, atrial natriuretic peptide, and LA size also did not exhibit any differences between the two groups. There were significant differences in the gender (68/88 [77%] males in the CM group and 35/58 [60%] in the UM group, OR = 2.23, 95% CI 1.08–4.61, *p* = .04). The type of AF also significantly differed between the CM and UM groups (*χ* ^2^ = 6.0466, df = 2, *p* = .049). The adjusted standardized residuals revealed that fewer patients had persistent AF (adjusted standardized residuals = 6.58) in the CM group (Table 1).

**TABLE 1:** Characteristics of the patients at baseline

Variable	CM group (*n* = 88)	UM group (*n* = 58)	*p* value
Age, year	66.7 ± 11.3	68.9 ± 8.5	.21
Male	68 (77)	35 (60)	.04
Body mass index, kg/m^2^	24.0 ± 3.7	24.6 ± 4.2	.16
Hypertension	60 (68)	38 (66)	.88
Diabetes mellitus	17 (19)	12 (21)	1
Chronic kidney disease			
G1	4 (5)	2 (3)	.14
G2	48 (55)	24 (41)	
G3a	19 (22)	19 (33)	
G3b	10 (11)	3 (5)	
G4	3 (3)	7 (12)	
G5	4 (5)	3 (5)	
Type of AF			.049
Paroxysmal AF	60 (68)	28 (48)	.01
Persistent AF	24 (27)	27 (47)	.005
Long‐standing AF	4 (5)	3 (5)	.62
CHA2DS2‐VASc score	3.0 ± 2.0	3.1 ± 1.7	.77
Antiarrhythmic drugs	58 (66)	16 (28)	<.001
BNP, pg/ml	99.3 (44.2–190.3)	144.4 (68.2–243.0)	.09
LV ejection fraction, %	64.0 (59.0–71.0)	63.0 (54.3–66.0)	.03
Cardiothoracic ratio, %	51.1 ± 6.1	52.2 ± 5.1	.21
LA volume (CT), ml	117.9 ± 46.9	127.3 ± 50.5	.33
LA diameter (TTE), mm	41.9 ± 6.1	43.2 ± 7.8	.26

Note

Categorical variables are expressed as the number (%). Continuous variables are expressed as the mean ± standard deviation and in the case of a skewed distribution, as the median and first‐third interquartile range (IQR).

Abbreviations: AF, atrial fibrillation; BNP, brain natriuretic peptide; CM, conventional method; CT, computed tomography; LA, left atrial; LV, left ventricular; TTE, transthoracic echocardiography; UM, high‐power and short‐duration ablation guided by unipolar signal modification.

### 3.2 Right pulmonary vein carina ablation and the anatomical features

**FIGURE 1:**
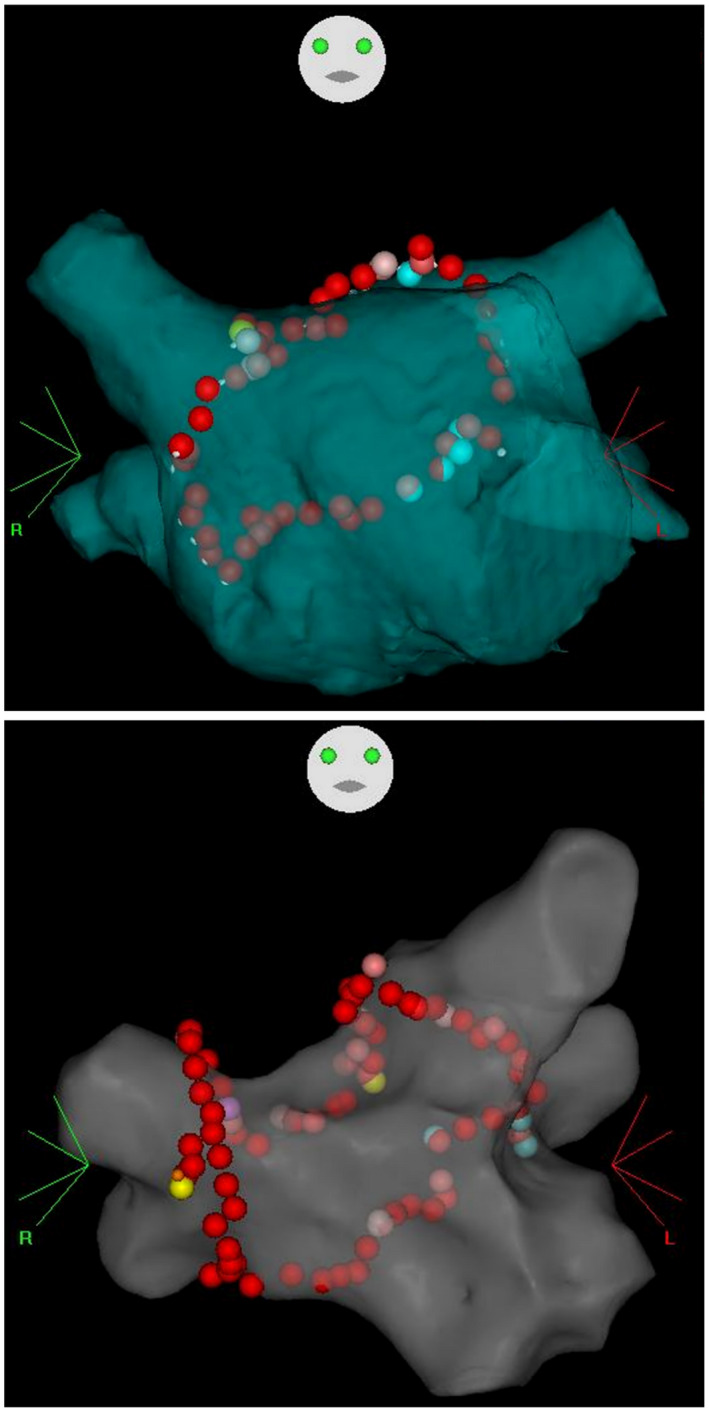
Ablation sites on a 3D CARTO map of the LA. A patient without an additional right PV carina ablation (Top) and a patient with an additional right PV carina ablation (Bottom) are shown. In the patient at the bottom, the pulmonary veins and LA were electrically isolated with an right PV carina ablation inside the first right PV ablation line. 3D, three dimensional; LA, left atrium; PV, pulmonary vein

In the CM group, fewer patients needed an additional right PV carina ablation than in the UM group (21/88 [24%] in the CM group and 30/58 [52%] in the UM group, OR = 0.29, 95% CI 0.14–0.60, *p* = .001) (Figure 1). Even if patients with initial ablation lesions applied on the right PV carina were included, the results were almost the same (27/94 [29%] in the CM group and 37/65 [57%] in the UM group, OR = 0.30, 95% CI 0.16–0.59, *p* < .001). No patients needed any RA ablation to achieve the right PV isolation. The number of right PVs and presence of structural heart disease were not associated with an additional right PV carina ablation. There were also significant differences in an additional roof line ablation (28/88 [32%] in the CM group and 32/58 [55%] in the UM group, OR = 0.38, 95% CI 0.19–0.75, *p* = .006). In contrast, an additional ablation at the region between the left PVs and LA did not exhibit any differences between the two groups (Table 2).

**TABLE 2:** Right PV‐RA connections and the anatomical factors

Variable	CM group (*n* = 88)	UM group (*n* = 58)	*p* value
Right PV carina ablation	21 (24)	30 (52)	.001
Number of right PVs			
2	63 (72)	37 (64)	.42
≧3	25 (28)	21 (36)	
Structural heart disease			
None	65	47	.86
Old myocardial infarction	5	2	
Coronary artery disease	9	4	
Nonischemic cardiomyopathy	8	4	
Valvular heart disease	0	0	
Tachycardiomyopathy	1	1	
Other epicardial connections			
The septopulmonary bundle	28 (32)	32 (55)	.006
Connections between the left PVs and LA through the LOM	21 (24)	13 (22)	1.00

Note

Categorical variables are expressed as the number (%).

Abbreviations: CM, conventional method; LA, left atrium; LOM, ligament of Marshall; PV, pulmonary vein; RA, right atrium; UM, high‐power and short‐duration ablation guided by unipolar signal modification.

### 3.3 Radiofrequency ablation parameters

The total procedure time was similar between the CM and UM groups (208.6 ± 45.5 vs. 196.3 ± 58.0 min, *p* = .48). The total RF ablation time was significantly longer in the CM group than UM group (3327 ± 882 vs. 2456 ± 852 s, *p* < .001). The average RF ablation time was longer in the CM group than UM group (31.0 ± 7.0 vs. 28.9 ± 4.9 s, *p* = .05). The number of RF ablation applications was significantly larger in the CM group than UM group (111 ± 34 vs. 85 ± 23, *p* < .001). With regard to the anterior aspect of the right PVs, the total RF ablation time and average RF ablation time were significantly shorter in the UM group than CM group (340 ± 73 vs. 511 ± 123 s, *p* < .001; 23.9 ± 3.2 vs. 38.7 ± 6.6 s, *p* < .001). The number of RF ablation applications did not differ between the CM and UM groups (Table 3).

**TABLE 3:** Procedural characteristics

Variable	CM group (*n* = 88)	UM group (*n* = 58)	*p* value
Total			
Procedural time, min	208.6 ± 45.5	196.3 ± 58.0	.48
Number of RF ablation applications	111 ± 34	85 ± 23	<.001
Total RF ablation time, s	3327 ± 882	2456 ± 852	<.001
Average RF ablation time, s	31.0 ± 7.0	28.9 ± 4.9	.05
Anterior side of the right PV			
Number of RF ablation applications	13 ± 3	14 ± 3	.3
Total RF ablation time, s	511 ± 123	340 ± 73	<.001
Average RF ablation time, s	38.7 ± 6.6	23.9 ± 3.2	<.001
Groin hematoma	1 (1)	0	1
Tamponade	1 (1)	2 (3)	.71
Periprocedural stroke	1 (1)	0	1
Esophageal fistula	0	0	1
Gastroparesis	1 (1)	1 (2)	1
Sinus rhythm at 1 year	66 (75)	45 (82)	.46

Note

Categorical variables are expressed as the number (%). Continuous variables are expressed as the mean ± standard deviation and in the case of a skewed distribution, as the median and first–third interquartile range (IQR).

Abbreviations: CM, conventional method; PV, pulmonary vein; RF, radiofrequency; UM, high‐power and short‐duration ablation guided by unipolar signal modification.

### 3.4 Procedural follow‐up

After a single ablation procedure, atrial arrhythmia recurrence at the 1‐year follow‐up was observed in 22 patients (25%) in the CM group and 10 (18%) in the UM group, respectively (*p* = .46). When adjusted for the age, gender, and AF pattern, the risk of recurrent atrial arrhythmias did not significantly differ between the CM and UM groups (HR = 0.60 for the UM vs. the CM group, 95% CI 0.2–1.4, *p* = .25). Subsequently, 16/22 (73%) patients in the CM and 6/10 (60%) in the UM group underwent a second procedure. In the CM group, 9/16 patients (56%) had reconnections of the box lesion, and only 1/16 (6%) had a reconnection of the right PV carina. In the UM group, 4/6 patients (67%) had reconnections of the box lesion, and 2/6 (33%) had reconnections of the right PV carina.

## 4 DISCUSSION

### 4.1 Main findings

The key observations of the present study were as follows. The RF ablation time was shorter in the UM group than CM group, while there were more patients who required an additional right PV carina ablation for the box isolation in the UM group than CM group. These results suggested that high‐power and short‐duration ablation guided by unipolar signal modification might be insufficient for ablating epicardial connections. In addition, there are three points to be discussed regarding the present study. First, local unipolar‐guided ablation is not suitable for a region with thick myocardium. Second, an epicardial connection on the right PV carina is a major anatomical impediment to creating a durable PVI. Finally, an acute transmural lesion can become a nontransmural lesion in chronic phase.

### 4.2 High‐power unipolar signal modification and the lesion transmurality

**FIGURE 2:**
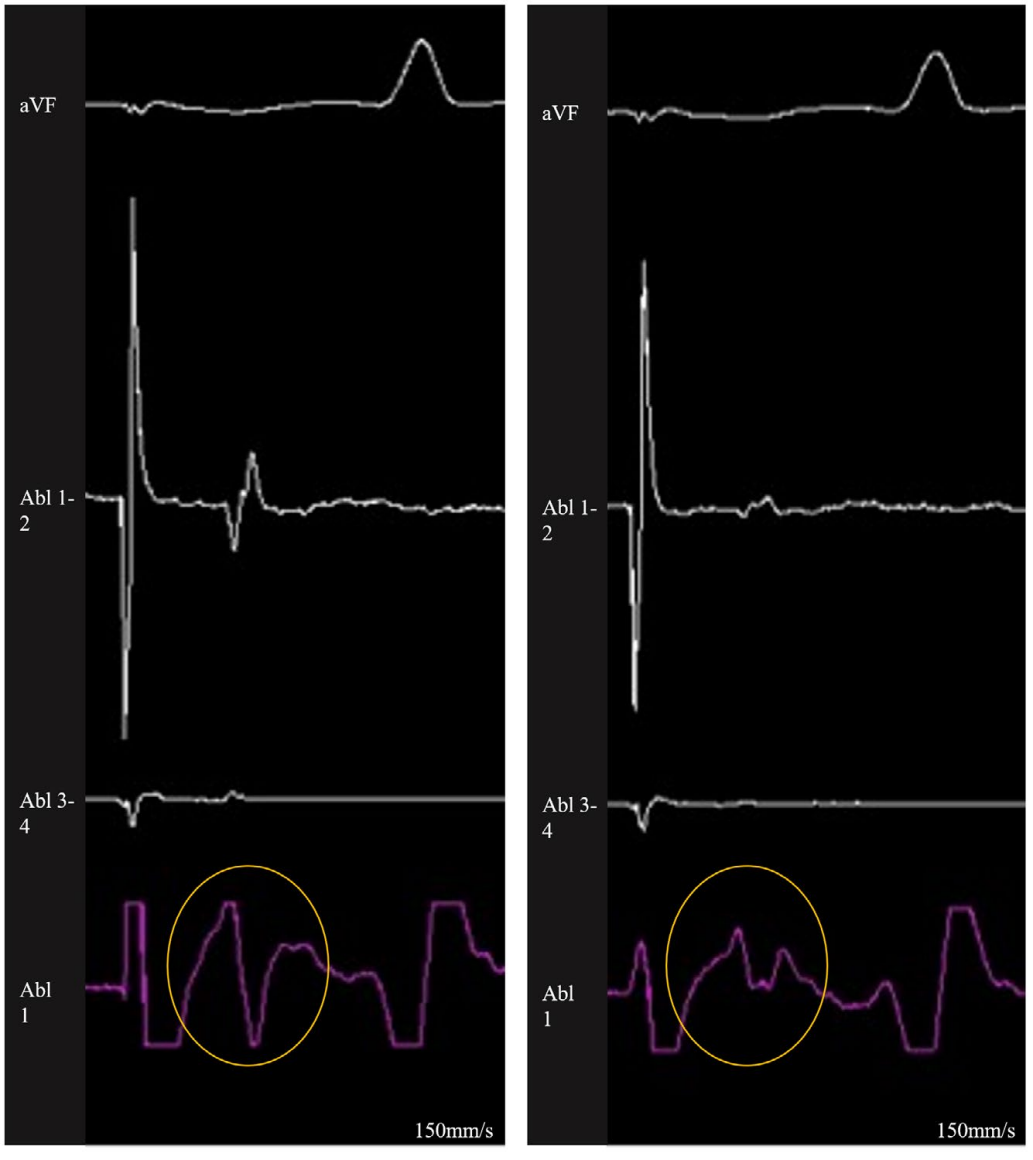
Unipolar atrial EGMs recorded before and after the ablation of the right PV anterior line in a case who required additional right PV carina ablation. This figure shows the intracardiac EGMs before (left panel) and after (right panel) the RF application. Surface ECG lead aVF, bipolar EGMs (Abl 1‐2 and Abl 3‐4), and unipolar EGMs (Abl 1) recorded by the ablation catheter are shown. The unipolar signal changed from a positive–negative morphology to a positive morphology (orange circle). ECG, electrocardiogram; EGMs, electrograms; PV, pulmonary vein; RF, radiofrequency

Hyperthermia causes myocardial damage, and thermal injury induced by electrical current deliveries with an ablation catheter is comprised of two components: resistive and conductive phases.^12^ The resistive heating reaches a maximum value a few seconds after starting the ablation, but the tissue necrosis is confined to within 1–1.5 mm from the electrode surface. In contrast, conductive heating increases slowly, and reversible tissue stunning can occur in a region far from the resistive heat source because the tissue temperatures drop after terminating the RF ablation application. Therefore, RF ablation requires at least 30–60 s to create irreversible lesions.^13^ A high‐power (40–50 W) increases the resistive heating size, and an in vitro porcine study demonstrated that high‐power short‐duration ablation with low irrigation flow rates created more superficial lesions with less heating of the deeper tissue layers than that observed with the conventional low‐power long‐duration ablation with high irrigation flow rates.^14^ On the other hand, the unipolar signal can provide a real‐time assessment of the lesion transmurality. When the electrical wavefront crosses healthy conductive tissue, the unipolar signal of the tissue displays a biphasic RS pattern and changes from an RS pattern to an R pattern in transmural lesions. During low‐power (25–30 W) ablation, the conductive phase is predominant at the epicardial aspect of the atrial myocardium, and a negative component abolition of the unipolar signal may correspond to a transmural but reversible lesion. In other words, a negative component abolition of the unipolar signal can occur before transmural necrosis is obtained.^15^ When we applied a high‐power (40–50 W) ablation, the resistive tissue lesion should predominate over the conductive lesion, and the negative component abolition of the unipolar signal and transmural necrosis can occur almost simultaneously. We should also note that in the experimental model, the unipolar signal criteria for the lesion transmurality were not validated at a site with a wall thickness of over 4 mm. In transmural lesions, a unipolar signal exhibits an elevation of the isoelectric line as well as a complete abolition of the negative deflection. In the region with epicardial connections with over a 4‐mm atrial wall thickness, a notch wave might remain in spite of an isoelectric line elevation and the disappearance of the negative deflection. This notch wave would show that there was an insufficient epicardial lesion (Figure 2). Additional work is necessary to confirm this hypothesis.^5^

Despite this advantage of high‐power using unipolar signal modification, on a thick atrial wall, the conductive phase predominates at the epicardial aspect of the atrial myocardium, and we should consider a prolonged RF energy delivery with other indices such as the change in the conduction time and sequence of the PV potentials, Ablation Index/Lesion Size Index, or change in the local impedance for creating a transmural necrosis. Larger, multicenter, prospective, and randomized studies are needed to determine the optimal index and ablation strategy for ablation of the right PV carina region.

### 4.3 Right pulmonary vein–right atrium connections and other epicardial connections

**FIGURE 3:**
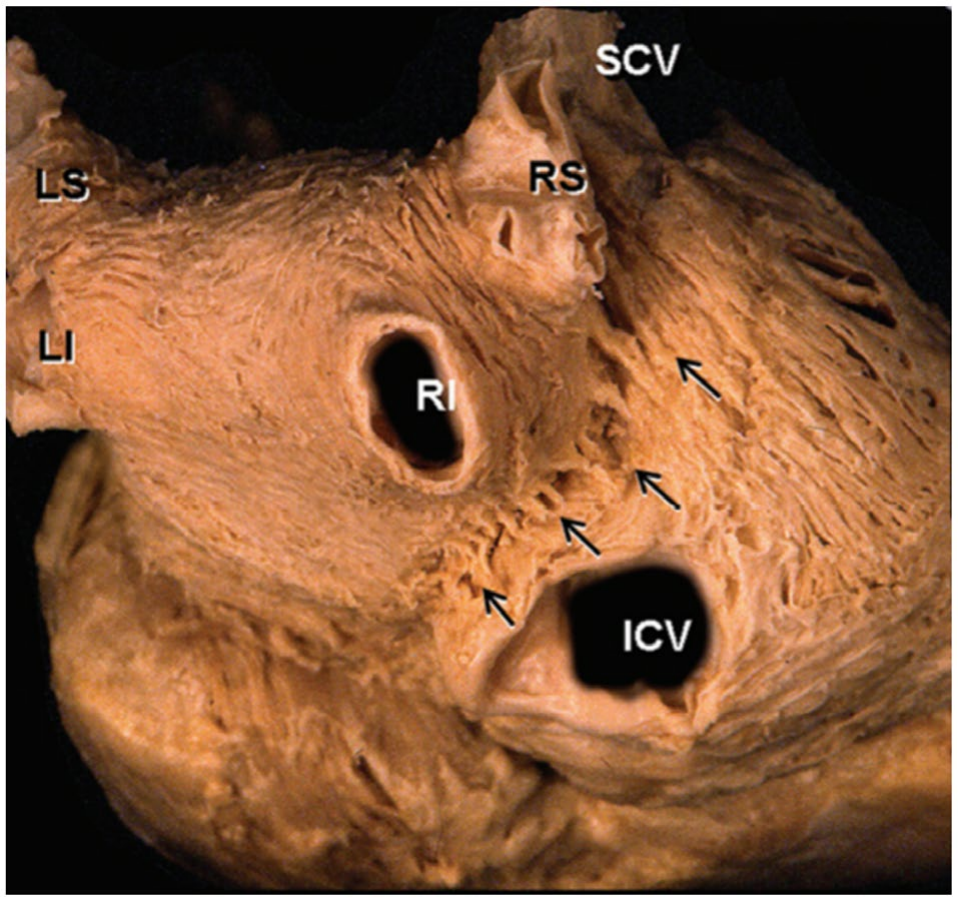
Gross anatomy of the epicardial connections between the right PV and RA. This dissection of the posterior and inferior parts of the interatrial groove shows multiple muscle bridges (arrows) connecting the LA and RA. ICV, inferior caval vein; LA, left atrium; LI, left inferior pulmonary veins; LS, left superior pulmonary veins; PV, pulmonary vein; RA, right atrium; RI, right inferior pulmonary veins; RS, right superior pulmonary veins; SCV, superior caval vein. Adapted from Ho et al^21^

Several earlier studies have focused on the carina region as an important ablation target in patients in whom a successful PV isolation was not achieved only by ablation encircling the PVs.^16^, ^17^, ^18^ A histological study found that the myocardial sleeves are the thickest in the inferior wall of the superior PVs and superior wall of the inferior PVs, indicating that this anatomical characteristic is a reason for a nontransmural ablation lesion adjacent to the carina and a failure of the PV isolation without a direct ablation targeting the carina.^19^, ^20^ Anatomical studies demonstrated epicardial connections between the right PVs and RA (Figure 3).^2^, ^21^, ^22^ It has also been reported that epicardial connections between the right PV carina and RA, as well as the thickness, lead to a nontransmural ablation lesion and require physicians to perform an additional carina ablation. In this past study, RF energy was delivered at a power of 25 to 30 W at the anterior aspect of the PV antrum, and a PVI was not achievable without a right PV carina ablation in one‐fifth of the patients.^23^ This energy is almost the same as that in the CM group. In the CM group, a box isolation was not achievable without a right PV carina ablation in one‐fourth of the patients. In contrast, almost twice as many patients in the UM group needed an additional right PV carina ablation as the patients in the CM group, probably because of an insufficient lesion depth.

Pambrun et al. reported that a high‐power (40–50 W) PVI displayed a higher first‐pass PVI than a low‐power (25–30 W) PVI although the RF duration was guided by unipolar signal modification in both groups.^6^ In an animal study, an extension of the RF ablation time after the elimination of the negative component of the local unipolar signal was critically important for creating a transmural necrosis. In the present study, RF ablation was delivered with 40 W and prolonged for 5 to 10 s after the elimination of the negative component in the UM group. The RF setting in the UM group seemed to be adequate for creating a transmural lesion. Therefore, the high‐power and short‐duration ablation guided by unipolar signal modification might be inappropriate for evaluating epicardial connections as in the right PV carina region. Kistler et al. also reported that a wide encirclement of the left and right PVs in pairs 1–2 cm from their ostia, as defined by PV angiography and the 3D map, was performed, and almost half of the cases needed additional RF ablation on the right intravenous ridge.^18^

**FIGURE 4:**
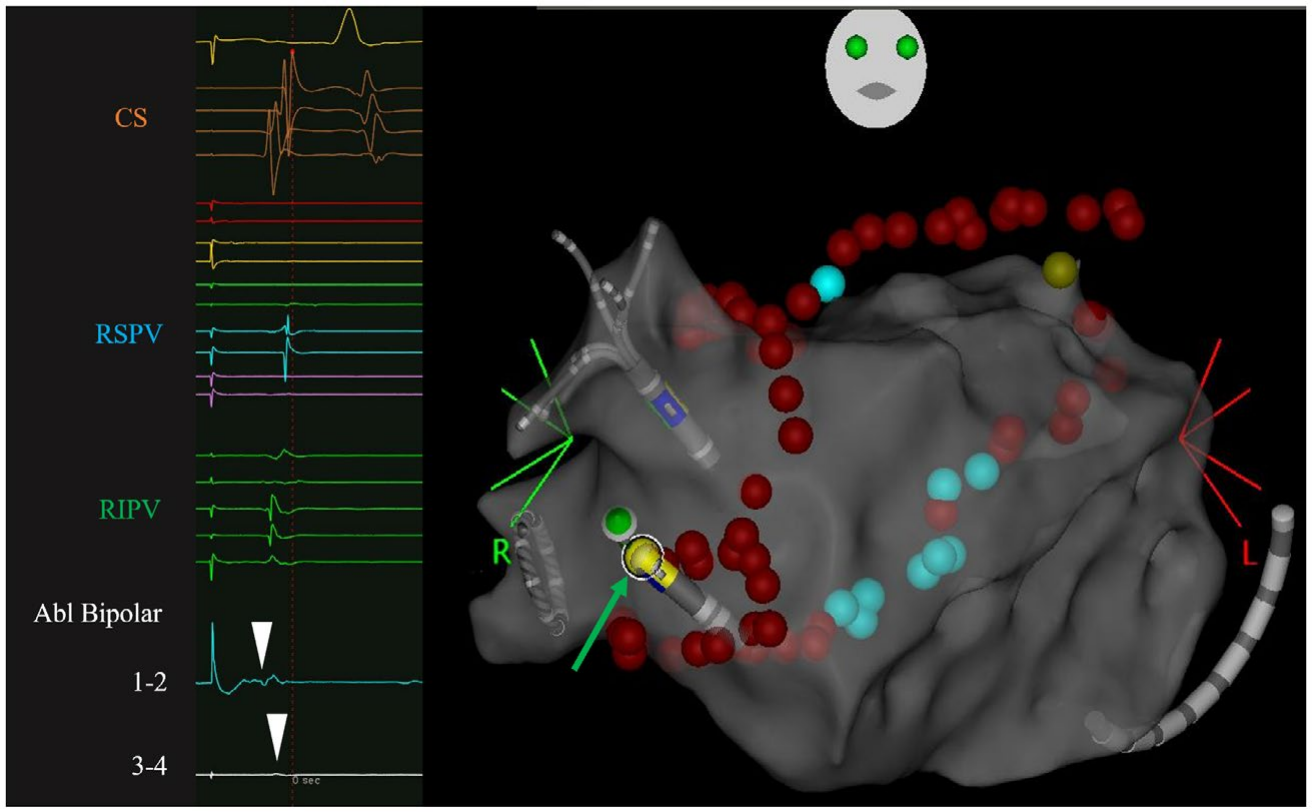
Ablation sites and local potentials on a 3D CARTO map of the LA. The ablation sites in the patient with an additional right PV carina ablation and successful site (green arrow) are shown. The local potentials at the success site (Abl Bipolar 1–2) were earlier than those near the right PV anterior line (Abl Bipolar 3–4). This is the same case as in Figure 2. 3D, three dimensional; LA, left atrium; PV, pulmonary vein; RA, right atrium

In the present study, there were no cases who required RF applications at an exit site on the RA posterior wall to achieve a box isolation as we previously reported.^11^ Instead of that phenomenon, when the earliest site was identified in the carina region far from the anterior line, we regarded that site as an entrance via an epicardial connection between the right PV carina and RA (Figure 4).

Other epicardial connections like the septopulmonary bundle and ligament of Marshall have also been reported.^10^, ^24^ The present study also suggested that high‐power and short‐duration ablation guided by unipolar signal modification might be insufficient for ablating the septopulmonary bundle. In the present study, we especially focused on right PV‐RA connections because of the following reasons. An ablation of the region of the right PV carina is a risk factor for phrenic nerve palsy and PV stenosis. In addition, right PV‐RA connections can be ablated from the RA.

### 4.4 Chronic reconnections of acute transmural lesions

Multiple studies have demonstrated a strong association between a durable PVI and the freedom from AF recurrence.^25^ Hansom et al. reported that patients undergoing high‐power short‐duration PVI ablation had a significantly higher rate of right PV carinal reconnections during redo procedures.^26^ In the present study, the number of patients who underwent a redo ablation was limited. However, more patients had reconnections of the box lesion through the right PV carina in the UM group (6%) than in the CM group (33%). In the region with epicardial connections like the right PV carina, the high‐power short‐duration ablation using unipolar signal modification might be associated with the failure to achieve chronic transmural lesions as well as acute transmural lesions.

### 4.5 Limitations

The limitations of our study were inherent to its retrospective observational nature, small number of patients, and single‐center study. Furthermore, we compared the groups treated during different periods. The technology of RF ablation has advanced, and the adaptation of RF ablation to AF has expanded over time. This progression might have led to the result that more patients had paroxysmal AF and were prescribed antiarrhythmic drugs in the CM group than UM group. However, patients with paroxysmal AF should have thicker atrial walls than those with nonparoxysmal AF.^27^

Furthermore, last but not least, we could not specify the contact force and RF duration (especially in the CM group) at each lesion. We used a steerable sheath in all the study patients, which facilitated an optimal contact force and catheter stability. In the UM group, the abolition of the negative deflection mostly occurred within 5 s, and we could then stop the RF application within 15 s. Thus we believe the ablation lesions in the UM group were created by resistive heating.

## 5 CONCLUSIONS

A box isolation was less achievable without RF ablation on the right PV carina in the UM group than CM group. We should consider RF ablation with a long‐duration for epicardial connections between the right PV carina and RA. Nevertheless, further larger, multicenter, prospective, and randomized studies are needed to confirm these results.

## CONFLICT OF INTEREST

Authors declare no conflict of interests for this article.
